# Health and social care use, costs, and satisfaction among key workers accessing Resilience Hub support during the COVID-19 pandemic

**DOI:** 10.1186/s12913-024-12066-w

**Published:** 2025-02-04

**Authors:** Aleix Rowlandson, Filippo Varese, Linda M. Davies, Paul French, Kate Allsopp, Lesley-Anne Carter, Daniel Hind, Katherine McGuirk, Alan Barrett, Gita Bhutani, Fay Huntley, Joanne Jordan, May Sarsam, Hein Ten Cate, Holly Walker, Ruth Watson, Jack Wilkinson, Jenni Willbourn, Gemma E. Shields

**Affiliations:** 1 School of Health Sciences, Faculty of Biology, Medicine and Health, Manchester Academic Health Science Centre, University of Manchester, Oxford Road, Manchester, M13 9PL UK; 2 Greater Manchester Mental Health NHS Foundation Trust, Manchester Academic Health Science Centre, Research and Innovation, 3rd Floor Rawnsley Building, Hathersage Road, Manchester, M13 9WL UK; 3 Faculty of Health, Psychology and Social Care, Manchester Metropolitan University, Brooks Building, Bonsall Street, Manchester, M15 6GX UK; 4 Greater Manchester Resilience Hub, Pennine Care NHS Foundation Trust, Trust Headquarters, 225 Old Street, Ashton-under-Lyne, Lancashire, OL6 7SR UK; 5 School of Health and Related Research, University of Sheffield, Regent Court, 30 Regent Street, Sheffield, S1 4DA UK; 6 Greater Manchester Health and Social Care Partnership, 4th Floor, 3 Piccadilly Place, Manchester, M1 3BN UK; 7 School of Health & Society, University of Salford, Mary Seacole Building, Frederick Road Campus, Broad St, Salford, M6 6PU UK; 8 Lancashire and South Cumbria Resilience Hub, Lancashire and South Cumbria NHS Foundation Trust, Sceptre Point, Sceptre Way, Walton Summit, Preston, PR5 6AW UK; 9 School of Psychology, University of Liverpool, Ground floor, Whelan Building Quadrangle Brownlow Hill, Liverpool, L69 3GB UK; 10 Cheshire and Merseyside Resilience Hub, Mersey Care NHS Foundation Trust, V7 Building, Kings Business Park, Prescot, L34 1PJ UK; 11 School of Health in Social Science, Old Medical School, University of Edinburgh, Teviot Place, Room 2.2, Doorway 6, Edinburgh, UK; 12 Humber and North Yorkshire Resilience Hub, West Park Hospital, Tees Esk and Wear Valleys NHS Foundation Trust, Edward Pease Way, Darlington, DL2 2TS UK; 13 Research and Innovation Department, Pennine Care NHS Foundation Trust, Trust Headquarters, 225 Old Street, Ashton-under-Lyne, Lancashire, OL6 7SR UK; 14 Division of Psychology and Mental Health, The University of Manchester, Manchester Academic Health Science Centre, Manchester, UK

**Keywords:** Key workers, COVID-19, Healthcare service use, Mental health, Service satisfaction

## Abstract

Key workers are vulnerable to adverse mental health. To ensure service sustainability during COVID-19, Resilience Hubs were established across England offering outreach, screening, and mental health support. This evaluation aimed to describe wider service use, associated costs, and satisfaction among Hub clients (key workers) accessing Hub support. Clients accessing support across four Hubs were invited to complete a service use questionnaire, between 5 and 8 months post referral, collecting satisfaction, wider service use and cost data. Exploratory linear regression assessed the relationship between demographic variables and service use. Most Hub clients reported Hub contact post referral (219/299, 73.2%), with many accessing mental health support (171/299, 57.2%) or on waitlists (34/299, 11.4%). Satisfaction was high, with median helpfulness rated 92 (out of 100), and many stating that Hubs either fully (148/299, 49.5%) or partially (54/299, 18.1%) met their needs. Mental health support accounted for most service use, with higher total service use and costs when including Hub services (£514 versus £213). Key workers have complex needs. Hub services helped clients to access support, with high reported satisfaction indicating that Hubs met clients needs. Further research is required to assess how service use varies according to occupation or demographic characteristics, and whether Hubs are clinically and cost-effective.

**Supplementary Information:**

The online version contains supplementary material available at 10.1186/s12913-024-12066-w.

## Background

Key workers are an important population, including, but not limited to, individuals from health, social care, emergency and education services, and other staff groups, such as the voluntary, community and social enterprise (VCSE) sector. Due to increased exposure to psychological/emotional stressors at work [1], key workers are particularly susceptible to adverse mental health, with increased rates of depression, anxiety, and post-traumatic stress disorder (PTSD) all noted [2–8].

Psychiatric morbidity not only affects key workers, but also service provisions, and society, with adverse mental health among this population previously linked to increased absenteeism and reduced quality of patient care [9]. A healthy workforce is therefore essential to ensuring the resilience and provision of key services. This is particularly important during times of emergency. However, emergency situations, notably the COVID-19 pandemic, often exert additional stress on key workers, with fears of exposure, transmission, and isolation all noted [10–12].

COVID-19 has been linked to increased adverse mental health among key workers [11, 13–17], with stress, anxiety, and depression reported as significantly higher than pre-pandemic values [15]. This posed a substantial threat to the resilience of key services. Additionally, several systemic and personal factors are noted as barriers to help seeking among key workers, including knowledge, and lack of service access and availability [18, 19]. As delayed help-seeking can contribute to increased duration of untreated illness and poorer prognosis, any perceived barriers among key workers can be seen to threaten the resilience of key services [20]. Recognising this and the need to support the mental health of frontline workers, several organisations and local systems established or repurposed services in the summer of 2020.

In autumn 2020, NHS England Improvement (NHSE/I) provided national funding to support mental health and wellbeing provisions [21], with 40 Resilience Hubs (also known as ‘Staff Mental Health and Wellbeing Hubs’) established or adapted across England. The Resilience Hubs aimed to provide mental health screening and facilitate access to psychosocial support for NHS, social care and emergency response staff affected by the pandemic [22, 23]. Alongside Hub establishment, a multi-site, mixed-methods study was conducted to evaluate the Hub model in four UK sites [24]. This included a health economic analyses of Hub clients’ service use data alongside more general satisfaction data, which are reported here.

Service use data is necessary to support economic analysis and provides useful information to justify resource allocation, plan for implementation, and enhance the sustainability of interventions and existing services. This was particularly important during COVID-19 which placed additional pressures on already limited healthcare resources, and also as psychiatric comorbidity is often associated with changes (generally increases) in healthcare resource use and costs [25]. However, despite the link between COVID-19 and increased psychiatric morbidity among key workers [11, 13–17], healthcare service use data was largely limited to general population samples [26, 27]. This provides limited evidence for decision makers trying to ensure the availability and sustainability of service provisions for key workers. Service use data among this population is therefore important to help assess the potential impact and feasibility of new interventions, such as the Hubs, and the level of service engagement. Meanwhile, data on client satisfaction, and the extent to which a new intervention meets individual needs can help demonstrate the putative functions of a novel intervention. This data not only supports the wider implementation of services, but also service improvement, helping to ensure that interventions, such as the Hubs, are well suited to clients’ needs.

### Aim

This research aimed to describe how a sample of clients (categorised as key workers) accessed Hub support and wider NHS and social care services, and the associated costs, during the COVID-19 pandemic. Client satisfaction with Hub services and the association between measured participant characteristics and total NHS and social care costs were also explored. These findings will inform our understanding of which services are most relevant to Hub clients and the benefits of Hub intervention for this population. Further, given the potential long-standing effects of the COVID-19 pandemic on mental health, it is crucial to identify whether Hub intervention is an acceptable form of support, and which other key supports are commonly utilised. This will help to inform future service offers and ensure effective funding allocation.

## Methods

### Resilience Hubs

This evaluation used data obtained between June 2020 and December 2021 from clients categorised as key workers accessing support across four Hubs in the north of England.

The included Hubs varied in terms of funding, design and populations served (further details are available in additional file 1 – Table 1, and are published elsewhere [28]). Three Hubs were newly established to support key workers, whilst one was pre-existing and expanded its provisions to include key workers (alongside other populations). All Hubs were NHS funded, though the specific source and amounts varied based on population size and weighted mental health need. Self-referral and mental health screening data informed subsequent clinical assessments across all Hubs. Assessments ranged from rapid assessments (via telephone) to in-depth assessment (via video consultation). Service provisions varied, ranging from onward referrals (to existing services) to direct provisions (of therapy/support). However, support offers were flexible, with Hubs adapting their provisions to the changing needs of clients (e.g., increasing outward referrals when working at maximum capacity). Most Hub support was provided virtually, with some expansion to face-to-face support as COVID restrictions eased [28].

Variability in support offers, both within and across Hubs, may be seen to influence both Hub support and wider service use costs, and is explored in more detail in the Results section.

All Hubs included in this evaluation routinely included research consent questions in their screening offers and used the mental health screening questionnaires to inform clinical assessments. This included measures of depression (PHQ-9), anxiety (GAD-7), post-traumatic stress (ITQ or PCL-5), and social and occupational functioning (WSAS), with some Hubs also including problematic alcohol use (AUDIT) [29–34]. An aggregate measure of overall severity (low, moderate, high) was used to summarise across the clinical measures collected during Hub screening, and this was defined by the highest severity categorisation received on any screening measure. Such a measure is important as mental illness severity has previously been linked to perceived treatment need, treatment contact and intensity, all of which may impact subsequent service use and associated costs [35, 36]. Demographic data (age, gender, ethnicity) was also routinely collected by the included services.

### Service use questionnaire

An online Service Use Questionnaire (SUQ) was administered to Hub clients between 5–8 months post screening, with data collected between March 2021 and March 2022. The SUQ was developed based on previously used health economic forms available from the project team [37], and asked participants to report any service use within the previous 6-months. This was developed with input from a patient and public involvement and experience (PPIE)/key worker consultation group (comprising Hub clients from sites A, B and D) who provided suggestions on content and usability (e.g., simplifying language; adding logic so relevant follow-up questions only show if particular responses are selected) prior to finalisation. The SUQ is available online via the Database of Instruments for Resource Use Measurement [38].

Psychiatric conditions can have physical manifestations which may impact service use [26]. Considering this, the SUQ intended to capture data on both mental health support (current, completed, discontinued/incomplete and waitlist) and wider service use (inpatient care, A&E, hospital visits and primary, community and social care). This included a series of pre-defined quantitative response questions (i.e., ‘Are you receiving any other types of mental health support?’, ‘How many appointments/sessions have been offered?’), and descriptive free-text response questions (i.e., ‘Please describe the support you are receiving and what type of service it is’). Details on the level of Hub support received, client satisfaction, and the extent to which services were accessed because of Hub support were also obtained.

### Participants

All Hub clients aged over 18 years, completed mental health screening 5–8 months prior, and consented to be contacted regarding follow-up research, were eligible for inclusion.

This included various key worker types, though local variation exists as some Hubs opened to different occupational groups in a phased approach (to avoid overwhelming Hub resources, or other clinical and support services). Key workers included health and social care staff, the ambulance service, police, and fire services, third sector services (social care; local authority-funded; private health and care) and voluntary, community and social enterprise staff. Some Hubs also included education staff (sites A and D), key worker family members (sites A, C and D), and younger health and care staff, aged 16–17 years (sites A, C and D).

### Recruitment and procedures

Eligible key workers were invited (via email) to complete the online SUQ approximately 5–8 months post screening. To reduce digital inequality, Hub clients who reported irregular email access or completed screening via telephone, were contacted via telephone and offered to complete the SUQ via telephone. To increase responses, up to four reminders were sent over 2 months until participants declined involvement or completed the measure. SUQ data were anonymised by Research Assistants at each Hub and compiled onto a central database. This database was managed by study statisticians and health economists who performed quality checks and re-coding/cleaning, in preparation for use.

### Ethics

Ethical approval was granted for the Resilience Hubs evaluation study through North West – Preston Research Ethics Committee IRAS Project ID 290,375 REC Reference 20/NW/0462. To protect Hub clients’ identity, all data were anonymised prior to analysis, with Hub sites also anonymised. Routinely collected screening data was only included in the analysis for Hub clients who gave consent to the use of their anonymised data for research purposes. Where the SUQ was completed online, proportionate consent was obtained. Where the SUQ was completed via telephone, informed consent was obtained verbally using a telephone consent form completed by the research assistant, with a copy mailed to the participating Hub client.

### Analysis

Screening data were analysed and summarised numerically, with an aggregate measure of symptom severity (low, moderate, high) used to outline the mental health needs of Hub clients at screening. Total scores across different measures were judged to determine the proportion of Hub clients meeting clinical thresholds for significant difficulties across the measured domains. These are reported in a separate paper. Data on mental health support access and satisfaction with Hub support were analysed using descriptive statistical summaries.

To aid analysis, service use descriptions were cleaned and recoded. Where Hub clients entered descriptions as free text, categories were collapsed by the research team to simplify analysis (e.g., “CBT” and “cognitive behaviour therapy” were collapsed into CBT). This allowed us to categorise results into key types of mental health support and to identify descriptions for unit costing.

Service use was costed from an NHS and social care services perspective using published standard national unit costs, including National Health Service (NHS) reference costs, the British National Formulary (BNF) and Personal Social Services Research Unit Costs of Health and Social Care [39–41]. Where published unit cost data could not be identified or were ambiguous, expert opinion (discussion with clinical experts from the research team and/or Hub staff) was used to derive unit cost estimates. All costs are reported in UK pounds sterling for the 2021 price year (available in additional file 1 – Table 2).

An exploratory linear regression analysis was conducted to assess whether measured participant characteristics (e.g., age, gender, symptom severity) were associated with total NHS and social care costs. Two separate models were run with mental health support and total costs (including wider healthcare) as outcomes. All analysis was conducted using Stata (Stata 14 (64-bit)), with a significance level of 0.05 used to interpret the results.

## Results

### Demographics

Screening data were obtained for 1,973 clients. However, only 900 clients who consented to be contacted for further research were eligible to be invited to complete the SUQ. Of these, 299 completed the service use measure, a response rate of 33.2%.

Participant characteristics are displayed in Table 1. Due to small numbers, demographic categories have been collapsed into broad categories (e.g., White British, or Black, Asian and minority ethnic) for presentation purposes. Despite some local variation, the sample was homogenous in terms of gender, ethnicity, and sexual orientation. With respect to mental health, clients were more heterogenous, with a large proportion demonstrating significant mental health/functional difficulties and domain scores suggestive of multiple co-morbid difficulties at screening.

**Table 1:** Demographic data for Hub service use questionnaire participants

Category	Mean (SD) / *n* (%)
**Age (yrs.)**	43.9 (10.5)
	*2 (0.7%) missing*
**Ethnicity**	
White British^a^	269 (89.9%)
Black, Asian and minority ethnic^b^	14 (4.7%)
Other	0 (0%)
* Missing*	*16 (5.4%) missing*
**Gender**	
Woman	247 (82.6%)
Man	43 (14.4%)
Identified in another way	5 (1.7%)
Prefer not to say	0 (0%)
* Missing*	*4 (1.3%) missing*
**Sexual orientation**	
Heterosexual	247 (82.6%)
Identified in any way other than heterosexual	27 (9.0%)
* Missing*	*25 (8.4%) missing*
**Disability**	
Yes	30 (10.0%)
No	261 (87.3%)
* Missing*	*8 (2.7%) missing*
**Occupation**	
Education	7 (2.3%)
Emergency	11 (3.7%)
Local authority	9 (3.0%)
NHS	153 (51.2%)
Other^D^	73 (24.4%)
Primary care	18 (6.0%)
Social care	9 (3.0%)
VCSE	9 (3.0%)
* Missing*	*10 (3.3%)*
**Overall symptom severity at screening**^**c**^	
Low	40 (13.4%)
Moderate	90 (30.1%)
High	165 (55.2%)
* Missing*	*4 (1.3%) missing*

^a^White British, white Irish and other white

^b^Black, Asian and minority ethnic includes Black African, Black Caribbean, other Black, Chinese, Indian, Bangladeshi, Pakistani, other Asian, White, and Asian, white and Black Caribbean, white and Black African, and other mixed

^c^This is an aggregate measure of overall severity, defined by the highest severity categorisation received on any screening measures

^D^In all sites other than Site D, free text information about job role were available, therefore it was often possible to re-categorise clients from ‘Other’ to one of the main reported categories included in the table, most commonly to the NHS category. However, this open text response option was not available for Site D, hence a high proportion of ‘Other’ job roles

### Hub further contact

Over two thirds of participants (219/299, 73.2%) reported Hub contact (of any kind) following referral. Fewer (171/299, 57.2%) reported receipt of mental health support (via any route). Among these, some employer provided services were cited (21/171, 12.3%). However, support was mostly either provided by Hubs (95/171, 55.6%), or accessed through Hubs (32/171, 18.7%), highlighting the important role of Hubs in service provision. Further specifics of Hub contact method and access route, stratified by site, are available in additional file 1 – Table 3.

### Satisfaction with Hub services

Table 2 displays client satisfaction with Hub services. High levels of satisfaction were observed, with median perceived helpfulness scored as 92 (out of 100). Most clients reported that Hub-delivered services either fully (148/299, 46.5%), or partially (54/299, 18.1%) met their needs, with only a minority (13/299, 4.3%) reporting unmet needs. More clients reported that the services accessed because of Hub support (onward referrals) were beneficial (83/299, 27.8%) than not (17/299, 5.7%). However, a minority (15/299, 5%) reported that Hub support with onward referrals was insufficient, which may have negatively impacted service access/engagement.

**Table 2:** Median (IQR) and n (%) pertaining to Hub satisfaction data

Category	Measure	Total (*n* = 299)
**How helpful was your contact with the Resilience Hub?**	Median (IQR)	92 (69–100)
	Min, Max	0, 100
	*Missing*	*95 (31.8%) missing*
**Did the Resilience Hub meet your needs?**	Yes, fully	148 (49.5%)
	Yes, partially	54 (18.1%)
	No	13 (4.3%)
	*Missing*	*82 (28.1%) missing*
**Did the Resilience Hub refer you to any other services/ help you to access any other services?**	1.Yes – The Hub helped me to access other services that I found beneficial	83 (27.8%)
	2. Yes - The Hub helped me to access other services, but it wasn’t quite the right service for me	17 (5.7%)
	3. No - I didn’t get enough help to access the support that I needed	15 (5.0%)
	4. No - They didn’t need to help me access other services as I got all the support, I needed directly from the Hub	67 (22.4%)
	5. No - I did not need any support from the Hub or referrals elsewhere	33 (11.0%)
	*Missing*	*84 (28.1%) missing*

### Support accessed and associated costs

Two hundred and thirteen (71.2%) participants reported sufficient detail to cost mental health support (i.e., fully reported the type of service and number of days/visits as relevant). For other health support (i.e., inpatient care, A&E, hospital visits, primary, community and social care), 237 (79.3%) reported sufficient details for costing. Subsequently, 182 (60.9%) of 299 participants reported sufficient data across both sections to estimate total service use costs. The sections below report service use and costs for participants with complete data (*n* = 182, 60.9%).

The mean time between screening and questionnaire completion was 7.85 months (SD 1.78, 95% CI 7.59–8.11). Table 3 summarises mental health support access among Hub clients. Note that as some clients reported multiple services and others reported no service use, percentages do not equate to 100%.

**Table 3:** Key types of mental health support among Hub clients with complete service use data (*n* = 182)

Key mental health service types	Number of participating Hub clients reporting, *n* (%)		
	Current	Complete	Incomplete
Bereavement support	1 (0.6%)	2 (1.1%)	0 (0%)
Counselling	11 (6.0%)	5 (3%)	1 (0.6%)
COVID-specific support^a^	1 (0.6%)	2 (1.1%)	0 (0%)
Digital interventions and support	0 (0%)	1 (0.6%)	0 (0%)
GP support	2 (1.1%)	1 (0.6%)	0 (0%)
Occupational health assessment & support	1 (0.6%)	1 (0.6%)	0 (0%)
Other third sector offer (charity)	1 (0.6%)	0 (0%)	0 (0%)
Other well-being support provided by the Hub^b^	4 (2.2%)	5 (2.7%)	1 (0.6%)
Peer support	1 (0.6%)	1 (0.6%)	0 (0%)
Pharmacological support	18 (9.9%)	2 (1.1%)	0 (0%)
Psychological therapy/support^c^	25 (13.7%)	26 (14.3%)	4 (1.1%)
Secondary care mental health support	1 (0.6%)	2 (1.1%)	0 (0%)
Well-being support	1 (0.6%)	1 (0.6%)	0 (0%)

^a^Including reported COVID support groups and clinics

^b^Note this applies to participants reporting non-specific Hub support, other support offers accessed via the Hubs will be included in the remaining categories (e.g., psychological therapy)

^c^Including all listed type of therapy, e.g., ACT, CAT, CBT, EMDR, IAPT services and more general descriptions (e.g., therapy and psychologist). The most common form of therapy reported was CBT

Only a minority of participants reported incomplete (i.e., discontinued) support. More clients reported current receipt of mental health support than completed support. This likely reflects the timing of SUQ completion and support accessed, including psychological therapy (25/182, 13.7%), pharmacological support (18/182, 9.9%), and counselling (11/182, 6.0%), which are typically delivered over an extended period. Given that symptom severity has previously been linked to treatment duration, this may also reflect high symptom severity at screening [35, 42].

Table 4 displays the mean service use and associated costs (to service providers) between screening and SUQ completion (7.85 months on average) among clients with complete service use data (*n* = 182). Low levels of service use were observed for inpatient (2/182, 1.1%), Accident and Emergency (A&E) (9/182, 4.9%) and community and social care (2/182, 1.1%), while use of hospital outpatient and day case services (21/182, 11.5%) were more frequently reported. Primary care services (61/182, 33.5%) were most cited, as would be expected.

**Table 4:** Categories of service use and associated costs among Hub clients with complete service use data (*n* = 182)

Category	Hub clients using a service, *n* (%)	Mean cost^a^ (95% confidence interval)		
		Total (including all services reported)	Excluding services delivered by the Hub^b^	Excluding services delivered by the Hub and services accessed due to Hub support^c^
**Mental healthcare**				
Current	52 (28.6%)	£204 (£141, £268)	£96 (£55, £136)	£54 (£25, £82)
Complete	44 (24.2%)	£164 (£108, £220)	£62 (£27, £98)	£24 (£3, £44)
Incomplete	5 (2.8%)	£8 (£0, £17)	£7 (<£1, £15)	£7 (<£1, £15)
Total mental healthcare		£376 (£294, £459)	£165 (£108, £221)	£84 (£47, £121)
**Wider health and social care**^**d**^				
Inpatient	2 (1.1%)	£34 (<£1, £80)	£34 (<£1, £80)	£34 (<£1, £80)
A&E	9 (4.9%)	£10 (£3, £18)	£10 (£3, £18)	£10 (£3, £18)
Hospital outpatient/day case	21 (11.5%)	£42 (£20, £65)	£42 (£20, £65)	£41 (£19, £64)
Primary care	61 (33.5%)	£47 (£19, £74)	£47 (£19, £74)	£40 (£12, £67)
Community and social care	2 (1.1%)	£5 (<£1, £11)	£5 (<£1, £11)	£5 (<£1, £11)
Total wider health social care		£138 (£73, £202)	£138 (£73, £202)	£129 (£64, £193)
**Total**		£514 (£410, £618)	£302 (£219, £386)	£213 (£140, £286)

^a^Mean costs borne by service providers associated with service use between screening and SUQ completion (7.85 months on average)

^b^Hub delivered mental health support excluded from costing. Note this information was not complete for all participating Hub clients and there may underestimate the cost of Hub delivered support

^c^Hub delivered mental health support and any services accessed because of Hub support excluded from costing. Note this information was not complete for all participating Hub clients and therefore may underestimate the cost of Hub delivered or accessed support

^d^Wider healthcare includes all healthcare visits (inpatient, A&E, hospital outpatient, primary care and community and social care). Hub phone calls reported separately in the initial questions were excluded from costing to prevent double counting, however, this may result in an underestimate

Owing to low levels of reported service use, mean costs were relatively low across categories. Total costs ranged from £213 excluding Hub services (both referred and delivered) to £514 including Hub services. Mental health support was the largest cost, ranging from 73.2% (£376/£514) when considering Hub services (both referred and delivered) to 56.6% (£165/£302) when excluding Hub provided services. However, the proportion of costs attributed to mental health decreased to 39.4% (£84/£213) when excluding all Hub services (delivered or accessed). This demonstrates that Hubs were a key driver of service use and associated costs among participants. When considering all Hub services, current (ongoing) mental health support was the largest cost driver across both mental health service use (54.2%, £204/£376) and total costs (39.7%, £204/£514). However, this could be expected as at the time of SUQ completion, Hub clients likely had ongoing needs (e.g., due to the ongoing pandemic). Wider health and social care use accounted for a minority of total costs (26.8%, £138/£514).

### Exploratory regression analysis

An exploratory linear regression was conducted to assess whether clients characteristics were associated with healthcare costs. Two models were run, using mental health and total costs (including wider health and social care) as outcomes. Using total costs, the regression model had an adjusted R-squared value of 0.103, indicating that the included characteristics were a poor predictor of total health and social care costs among participants. This also suggests that total costs are more affected by unmeasured covariates.

Table 5 displays the outcomes for the regression model investigating the association between clients characteristics and mental health costs. The regression model had an adjusted R-squared value of 0.235 (versus 0.103 for total costs), indicating that mental health costs were more likely related to the measured characteristics. As would be expected, having an emotional wellbeing concern prior to the COVID-19 pandemic was significantly associated with mental health costs (coef. 213.76, *p* = 0.035). Hub accessed was also significantly associated with mental health costs when comparing sites A and D (coef. 632.19, *p* < 0.001), likely reflecting differences in symptom severity, service availability, provisions, and length of follow up.

**Table 5:** Linear regression with mental health support costs (dependant variable) and Hub clients characteristics

Mental health support	Coefficient	Standard error	*P* value	95% confidence interval
Age	−7.94	4.24	0.063	−16.32, 0.44
**Gender**^**a**^				
Man	127.14	117.83	0.282	−105.60, 359.88
Another way	476.48	438.77	0.279	−390.17, 1343.14
**Black**,** Asian and minority ethnic groups**	−252.74	201.88	0.212	−651.5, 146.02
**Sexual orientation**^**b**^	−10.75	153.47	0.944	−313.87, 292.38
**Disability**^**c**^	36.26	176.48	0.837	−312.33, 384.85
**Emotional wellbeing concern before COVID**^**d**^				
Yes	213.76	100.41	0.035	15.44, 412.08
Unsure	−88.93	133.89	0.508	−353.38, 175.52
Impact of COVID-19				
Bereavement	−129.38	116.93	0.270	−360.33, 101.57
Undertaking new tasks within usual role	120.83	87.21	0.168	−51.42, 293.09
Suffered financial loss within the household	−128.40	113.82	0.261	−353.21, 96.42
Ill with COVID-19 (including being in hospital)	145.65	234.93	0.536	−318.38, 609.68
Family member ill with COVID (recovered at home)	64.86	111.19	0.560	−154.76, 284.48
**Overall symptom severity**^**e**^				
Moderate	236.73	126.76	0.064	−13.64, 487.1
High	207.89	121.81	0.090	−32.71, 448.49
**Registered Hub**^**f**^				
Hub A	632.19	115.82	0.000	403.41, 860.96
Hub B	235.03	141.85	0.100	−45.14, 515.21
Hub C	261.56	149.03	0.081	−32.8, 555.91
Constant	269.96	377.07	0.475	−474.81, 1014.74

^a^Reference case ‘women’ used to compare against other reported gender options

^b^Reference case ‘not heterosexual’

^c^Reference case ‘no disability’

^d^Reference case ‘no’

^e^Reference case ‘low’

^f^Reference case ‘Hub D. *n*=176/299 participants, R-squared =0.314, Adj R-Squared = 0.235

## Discussion

SUQ data was collected from a subsample of 299 Hub clients. Of these, 219 (73%) reported at least some contact with Hubs following initial screening, 171 (57%) reported accessing mental health support (via any route) since screening, and 34 (11%) reported being on a waiting list for mental health support. Three quarters of respondents who had accessed mental health support since screening did so because of their involvement with the Hubs. Survey respondents reported high levels of satisfaction with Hub support, with many reporting that the Hubs either fully (148/299, 49.5%) or partially (54/299, 18.1%) met their needs.

Service use appeared low, especially when considering the mental health needs of Hub clients. The cost analysis demonstrates that services delivered or accessed because of Hub support made up over half of the total health and social care service costs. In a group with a high mental health need, this might suggest that Hubs had a positive impact on ensuring access to services. Whilst it cannot be concluded with certainty (as we do not have a comparator arm), given the impact of COVID-19 on existing mental health services, it is very unlikely that service use cost would have been similar in the absence of Hubs. Wider service use remained relatively stable across categories, indicating that Hub services have little influence. However, as some clients were still in receipt or on waiting lists for support, final service use and associated costs are likely to have been higher than those reported.

Previous service use and cost estimates are available from randomised controlled trials of mental health populations within the UK [43–49]. While heterogeneity in terms of populations characteristics, time frames, and costing (e.g., included components, perspective, etc.) precludes a formal comparison, initial results suggest that service use and associated costs among Hub clients was comparatively low, especially when considering the mental health needs reported at screening (55% of clients having a high symptom severity classification at screening).

The potentially limited service use observed among Hub clients may be attributed to several individual or systemic factors. Previous research suggests that key workers may avoid help-seeking to avoid adding pressure on their colleagues, or services [50, 51]. Both an ‘awareness of burden on colleagues and patients’ and being ‘worried about imposing on another busy doctor’ have previously been cited as barriers to healthcare among physicians [51]. Perceived stigma surrounding mental health is also commonly cited, with fears surrounding negative career impact, being perceived as ‘weak’, ‘unfit to work’ or ‘unable to cope’ previously noted within post disaster/emergency evidence [18, 52–54]. Research among Hub clients, described elsewhere, also corroborated these findings with factors such as stigma, negative workplace cultures, and negative beliefs regarding workplace stressors cited as barriers to support seeking [55]. However, the access routes for Hub support in which clients can self-refer (thereby avoiding workplace involvement) may have helped to promote uptake of services. For instance, while around half of clients (51%) accessed Hub support following an email from their workplace, many were informed and accessed support through other channels (i.e., word of mouth, social media or online sources, email from Resilience Hubs). Potentially, observed service use may have been lower without the self-referral option for Hub services.

Lower service use may also reflect the occupational status of Hub clients, who often work long and irregular hours [55]. Pre-pandemic research noted ‘lack of time’ as the greatest barrier to help-seeking among doctors seeking help for stress/burnout [56], with time also noted as a key barrier during COVID-19 [57]. Given that 71% of NHS staff reported working overtime during COVID-19 [52], it is likely that lack of time may have impeded healthcare access among our sample. This is particularly relevant as over 50% of participating Hub clients were NHS employees. Additionally, the timing of data collection, between March 2021 and March 2022, meant that the recall period for some participants is likely to have been impacted by reduced healthcare service availability observed during and after lockdown periods [26].

The pandemic saw decreased availability of, and referrals to, healthcare services, tied to both staff redeployment and lockdown restrictions [58–60]. UK research reported significant reductions in mental health referrals over the initial lockdown period, followed by a gradual increase following the easing of restrictions (when service availability resumed) [60, 61]. Related, the timing of SUQ completion may have played an important role in reported service use, with many Hubs reporting ongoing increases in referrals since the time of data collection. This could be linked to increases in awareness of service provisions and trust in service offers, as well as key workers having more time to access services when less affected by the pandemic. Furthermore, evidence of delayed dysfunction (in which symptoms present later) following previous disasters suggests that the mental health needs of clients may not have peaked at the time of questionnaire completion, contributing to lower levels of service use than would otherwise be expected [62]. It is important to note that this demonstrates that the Hubs appear effective in ensuring a population in need access appropriate support services despite facing multiple barriers, and in a time with vast service disruption.

Exploratory regression did not find any Hub clients measured characteristics to be significantly associated with total costs. However, having a mental health concern prior to the COVID-19 pandemic was significantly associated with mental health costs. Although somewhat limited, this highlights the importance of obtaining screening data for the purpose of resource planning. This may be particularly useful to future decision makers when looking at which factors to account for when allocating funds. When comparing sites A and D, the site of Hub support was significantly associated with mental health costs, although differences were not significant across other Hub sites. While this likely reflects differences in populations serviced, client needs, and service availability, this also highlights variability across Hubs, emphasising the importance of decision makers adopting an individualised approach when considering funding and service delivery models.

While efforts were made to ensure that our research is robust and reliable, some limitations exist. Of the 900 Hub clients who consented to research follow up invited to participate, only 299 (33.2%) completed the SUQ. When considering the 1973 Hub clients who completed screening across the Hubs, this number represents an even smaller proportion of Hub users. Reduced participation was not unique to this research, with many trials also reporting reduced recruitment/retention during COVID-19 [63]. However, low recruitment and attrition can compromise the validity of research conclusions. Despite this, population characteristics between the sample of Hub clients with complete SUQ were largely comparable to the original sample of 1973 clients who initially completed screening in terms of age, gender, ethnicity and disability (details of which are published elsewhere) [64]. As Hub clients chose to complete the SUQ when invited, there is also a risk of self-selection bias. While all outcomes could be affected, satisfaction data was at particular risk (i.e., as clients with positive Hub experiences may be more likely to engage with Hub research). The sample was also largely homogenous across several key characteristics (e.g., gender, ethnicity, occupational status), reducing external validity for key workers with dissimilar characteristics and preventing subgroup analysis. However, the observed homogeneity may also reflect that women represented three-fifths of all key workers in the UK during COVID (58%) [65], or previously established patterns and barriers to help seeking among different genders and ethnic groups [66, 67]. A gender gap in mental well-being decline in the UK at the onset of the Covid-19 pandemic has also been established, which may also help explain the disproportionate representation of females [68]. As service use was reported retrospectively (i.e., they were asked to report any service use since screening), there was an increased risk of recall bias (i.e., clients may have forgotten using a service), increasing uncertainty surrounding key findings. However, self-report data was preferred over administrative data collected at sites as this ensured to capture wider service use, not always included within administrative datasets. The cross-sectional nature of this research and lack of pre-pandemic baseline data prevents comparison, and it is therefore difficult to measure/assess the true impact of Hubs on service use. It is also likely that reported service use may have been impacted by the timing of questionnaire completion, with service use likely influenced by the evolving nature of the pandemic (i.e., less services were available during lockdown periods [27, 60, 69]). While our analysis did not account for temporal trends, client’s mental health difficulties will have also likely varied over the pandemic (i.e., as COVID-19 related stressors increase/wain), impacting both the level of need and service use [70]. The observational nature of this research prevents service use from being tied to wider health outcome data (i.e., changes in depression scores), slightly limiting the usefulness of findings. The evolving nature of Hub support also limited our ability to include and comprehensively evaluate all service offers (i.e., as team support was expanded after our research had been commissioned, Hubs did not have permission/infrastructure to gather data on team support clients). Hubs were also variable in terms of their support, often flexing, and adapting service provisions to help meet the changing needs of populations throughout the pandemic. For instance, all Hubs incorporated the provision of direct therapy within their service models in response to local contexts (i.e., extensive waiting lists within external services), with full details reported elsewhere [28]. However, service heterogeneity makes it more challenging to formally assess the impact of Hubs on service use (i.e., increased engagement with external services may be more reflective of a Hubs delivery model, rather than a client’s increased desire to access additional services).

This evaluation has demonstrated the feasibility of obtaining service use and mental health screening data across a limited number of Hubs. However, future research would be greatly facilitated through the standardisation of both screening and outcome data across all Hub sites in England. Screening should obtain data on the demographic, occupational and mental health characteristics of clients, alongside measures which are useful for economic evaluation (e.g., service use, EQ-5D), and consent for both anonymous data use and to be contacted for future research. This, when combined with appropriate longitudinal follow up data (e.g., service use and mental health outcomes) would provide an opportunity to explore a range of relevant questions (i.e., how Hub support influences clients’ healthcare service use compared to baseline). However, follow-up data would need to either be time-bound to a clients’ registration with Hubs (e.g., six months post screening), obtained at the end of intervention/service delivery, or at discharge, to enable meaningful comparison. As our sample was largely homogenous in respect to several key demographic and occupational characteristics (e.g., gender, ethnicity, etc.), this not only limited our ability to conduct subgroup analysis, but also the external validity of our findings. Future research would greatly benefit from efforts to obtain and analyse data on a more diverse sample. Certain characteristics (e.g., ethnicity, gender, occupation) may influence both an individual’s health risks, and the ways in which they interact with services. It is therefore important to capture data on a diverse range of individuals so that we can better understand how their service use/needs may differ. This can help services to effectively plan and allocate resources, and appropriate outreach (e.g., where service use may indicate barriers to access among specific populations). Earlier and more extensive engagement (e.g., involvement with research design) with key workers from more diverse populations may help to ensure that research questions and methods are meaningful and accessible for a wide range of individuals.

Questions around the clinical and cost-effectiveness of Hub support remain. Future research should therefore focus on collecting sufficient data (including both clinical and health economic) to enable a robust economic evaluation of Hub services. While outcomes could be based on some of the measures already employed within this evaluation, steps could be taken to increase the robustness of findings (e.g., obtaining medical record data to supplement patient reported service use; ensuring that follow-up is long enough to capture all relevant outcomes; obtaining sickness/absence data directly from employers). This would provide useful evidence for decision makers in the NHS and could be used to support further promotion/investment in Hub services.

## Conclusion

Hub clients, comprising various key worker categories, accessing Hub support during the COVID-19 pandemic had complex care needs. However, when considering the mental health needs of Hub clients at screening, observed service use appeared low. Mental healthcare accounted for most of the observed service use and costs, with Hub support identified as a gateway to accessing other support services which may not otherwise have been accessed. This suggests that as a single point of access, Hubs act as a conduit to navigating the many other expert resources available, utilising a trusted assessor pathway model. High levels of satisfaction were also reported among clients accessing Hub services, suggesting that Hub services were effective at meeting the needs of key workers. Future research should focus on capturing service use data from a larger, more heterogenous population, to help draw meaningful comparison on how service use can vary based on key occupational and demographic characteristics.

## Supplementary Information

Supplementary Material 1.

## Data Availability

The data that support the findings of this study are not openly available due to reasons of sensitivity and are available from the corresponding author upon reasonable request. Data are located in controlled access data storage at The University of Manchester.

## Abbreviations

A&E: Accident and emergency
ACT: Acceptance and commitment therapy
AUDIT: Alcohol Use Disorders Identification Test)
BNF: British National Formulary
CBT: Cognitive behavioural therapy
EMDR: Eye Movement Desensitization and Reprocessing
GAD-7: General Anxiety Disorder-7
IAPT: Improving Access to Psychological Therapies
IQR: Inter quartile range
ITQ: International Trauma Questionnaire
NHS: National Health Service
NHSE/I: NHS England Improvement
PCL-5: Posttraumatic Stress Disorder Checklist for DSM-5
PHQ-9: Patient Health Questionnaire-9
PPIE: Patient and public involvement and experience
PSSRU: Personal Social Services Research Unit
PTSD: Post-traumatic stress disorder
SUQ: Service use questionnaire
WSAS: The Work and Social Adjustment Scale
