# Supplementary material for: Health and social care use, costs, and satisfaction among key workers accessing Resilience Hub support during the COVID-19 pandemic
Source: BMC Health Serv Res. 2025 Feb 4;25:205. doi: 10.1186/s12913-024-12066-w (PMC11796002; doi:10.1186/s12913-024-12066-w)
Supplement: Supplementary file 1 — Supplementary Material 1. [file 12913_2024_12066_MOESM1_ESM.docx]

**Resilience Hubs service use paper additional file 1**

Table 1: Groups in scope for Hub support at each site

|  | **Site A** | **Site B** | **Site C** | **Site D** |
| --- | --- | --- | --- | --- |
| **Over 18** (health & care staff) | ✓ | ✓ | ✓ | ✓ |
| **16-17 years** (health & care staff) | ✓ | Not in scope | ✓ | ✓ |
| **Family members*** | ✓ | Not in scope | ✓ | ✓ |
| **Ambulance**  **Service** | ✓ | ✓ | ✓ | ✓ |
| **Police/Fire** | ✓ | ✓ | ✓ | ✓  (if involved in specific COVID-related health & care duties) |
| **Education** | ✓ | Not in scope | Not in scope | ✓  (if responsible for wellbeing) |
| **3^rd^ Sector**** | ✓ | ✓ | ✓ | ✓ |
| **VCSE** | ✓ | ✓  (if local authority commissioned) | ✓ | ✓ |
| *Definition of family members differed across sites. Within Site C and Site D, family members referred to both immediate and chosen family living in any location. Within Site A, family members referred to immediate family, including in-laws, who must live in the Hub’s region.  ** Social care; local authority-funded; private health and care | | | | |

Table 2. Unit costs of health and social care used in costing analysis

| **Service type** | **Unit cost** | **Unit measure** | **Reference** |
| --- | --- | --- | --- |
| **Mental health unit costs** | | | |
| AA | £0.00 | NA | Assumption (cost is negligible based on service data). |
| ACT | £91.60 | Per contact | PSSRU Unit Costs of Health and Social Care 2021. Scientific and professional staff (Band 7, 8A, 8B, 8C, 8D). |
| Alcohol team | £92.28 | Per contact | NHS reference costs 2019/2020 updated to 2021 prices. |
| Care coordinator | £54.00 | Per contact | PSSRU Unit Costs of Health and Social Care 2021. Community based scientific and professional staff (band 6). |
| CAT | £91.60 | Per contact | PSSRU Unit Costs of Health and Social Care 2021. Community based scientific and professional staff (Band 6, 7). |
| CBT | £108.76 | Per unit | PSSRU Unit Costs of Health and Social Care 2017. Cognitive behavioural therapy (CBT) 6.7 pg 89. Using the last CBT cost published by PSSRU inflated to 2020-21 prices |
| CMHT | £38.00 | Per contact | PSSRU Unit Costs of Health and Social Care 2016 updated to 2021 prices. Community mental health team for adults with mental health problems. |
| Coaching | £81.00 | Per contact | PSSRU Unit Costs of Health and Social Care 2021. Scientific and professional staff (Band 6). Assumed 1.5 hour duration. |
| Cognitive assessment/ screening | £116.96 | Per initial assessment | NHS reference costs 2019/2020 updated to 2021 prices. |
| Counselling (including general and bereavement) | £59.00 | Per contact | PSSRU Unit Costs of Health and Social Care 2021. Scientific and professional staff (Band 6, 7). |
| Covid support group | £7.35 | Per attendance | PSSRU Unit Costs of Health and Social Care 2021. Scientific and professional staff (Band 8A, 8B, 8C, 8D), with a 1.5 hour duration and 20 attendees (cost is per person attending). |
| Crisis phone line | £0.00 | NA | Assumption (staffed by volunteers). |
| Crisis team | £97.85 | Per contact | NHS reference costs 2019/2020 updated to 2021 prices. |
| EMDR | £147.00 | Per contact | PSSRU Unit Costs of Health and Social Care 2021. Scientific and professional staff (Band 8A, 8B, 8C, 8D). 90 minute duration assumed. |
| Home treatment team | £23.00 | Per contact | PSSRU Unit Costs of Health and Social Care 2021. Community based social care professionals. |
| Hub support (type not specified) | £17.50 | Per contact | PSSRU Unit Costs of Health and Social Care 2021. Community based social care professionals (Band 4). 0.5 hour duration assumed based on hub feedback. |
| IAPT^1^ | £125.48 | Per contact | NHS reference costs 2018-19 (used as 2019-20 does not have a number of episodes), inflated to 2020/2021 prices. Using the greater need gives a lower cost, so the same cost has been used for all IAPT services to prevent confusion. |
| Long covid programme | £158.63 | Per attendance | NHS reference costs 2019/2020 updated to 2021 prices (assumed equal to a rehabilitation outpatient attendance). |
| Mind charity support | £0.00 | NA | No unit cost available. |
| Occupational health | £75.25 | Per attendance | NHS reference costs 2019/2020 updated to 2021 prices. |
| Online chat support | £0.00 | NA | No unit cost available. |
| Peer support | £12.50 | Per attendance | PSSRU Unit Costs of Health and Social Care 2021. Community based scientific and professional staff. Assumed 8 people in attendance for 1 hour session delivered by band 7 staff with support from band 4 (based on communication with services). |
| Pharmacological^a^ | £354.05 | Per 6-month duration | British National Formulary 2022 (weighted average of all Sertraline 50mg tablet with typical daily dose assumed). |
| Pharmacy | £4.77 | Per consultation | PSSRU Unit Costs of Health and Social Care 2021.Community-based scientific and professional staff. |
| Phone support (unspecified) | £125.48 | Per contact | IAPT cost assumed. NHS reference costs 2018-19 (used as 2019-20 does not have a number of episodes), inflated to 2020/2021 prices. Using the greater need gives a lower cost, so the same cost has been used for all IAPT services to prevent confusion. |
| Psychiatrist | £123.00 | Per contact | PSSRU Unit Costs of Health and Social Care 2021.Hospital based doctors. |
| Psychologist | £102.00 | Per contact | PSSRU Unit Costs of Health and Social Care 2021.Community based scientific and professional staff (Band 7, 8A, 8B, 8C, 8D, 9). |
| Sleep station | £0.00 | NA | No unit cost available (CBT based insomnia programme, delivered through an application). |
| Smartphone apps | £0.00 | NA | Assumption (smartphone applications cited ‘Mindfulness’ and ‘Oak’ free to download and use). |
| Spiritual guidance | £41.00 | Per contact | PSSRU Unit Costs of Health and Social Care 2021. Hospital based chaplain staff (Band 5). |
| Therapy (high intensity) | £121.33 | Per contact | PSSRU Unit Costs of Health and Social Care 2021. Community based scientific and professional staff (Band 8B, 8C and 9). |
| Trauma CBT | £98.00 | Per contact | PSSRU Unit Costs of Health and Social Care 2021. Community based scientific and professional staff (Band 8A, 8B, 8C, 8D). |
| Trauma EMDR | £98.00 | Per contact | PSSRU Unit Costs of Health and Social Care 2021. Community based scientific and professional staff (Band 8A, 8B, 8C, 8D). |
| Wellbeing support | £35.00 | Per contact | PSSRU Unit Costs of Health and Social Care 2021. Community based scientific and professional staff (Band 4). |
| **Inpatient** | | | |
| Foot fracture nonelective inpatient | £3,770.67 | Per stay (non-elective long stay) | NHS reference costs 2019/2020 updated to 2021 prices (weighted average of all foot fracture procedures) |
| Liver issue nonelective inpatient | £3,402.15 | Per stay (non-elective long stay) | NHS reference costs 2019/2020 updated to 2021 prices (weighted average of Liver failure disorders, Percutaneous Ablation of Lesion of Liver or Pancreas, Percutaneous Trans vascular Biopsy of Lesion of Liver, Percutaneous punch Biopsy of Lesion of Liver, Chemoembolization or Radioembolization, of Lesion of Liver). |
| Nasal polypectomy elective inpatient | £2,903.34 | Per stay (elective inpatient) | NHS reference costs 2019/2020 updated to 2021 prices. |
| Surgery elective inpatient | £3,740.73 | Per stay (elective inpatient) | NHS reference costs 2019/2020 updated to 2021 prices (weighted average of all descriptions citing 'surgery' or 'surgical', excluding those for populations aged ≤18 years) |
| Surgery nonelective inpatient | £3,194.22 | Per stay (non-elective long stay) | NHS reference costs 2019/2020 updated to 2021 prices (weighted average of all descriptions citing 'surgery' or 'surgical', excluding those for populations aged ≤18 years) |
| Pneumonia nonelective inpatient | £2,929.54 | Per stay (non-elective long stay) | NHS reference costs 2019/2020 updated to 2021 prices (weighted average of all descriptions citing pneumonia). |
| Sepsis nonelective inpatient | £3,405.38 | Per stay (non-elective long stay) | NHS reference costs 2019/2020 updated to 2021 prices (weighted average of all descriptions citing sepsis). |
| Gastrointestinal bleed nonelective inpatient | £2,392.93 | Per stay (non-elective long stay) | NHS reference costs 2019/2020 updated to 2021 prices (weighted average of all descriptions citing gastrointestinal bleed). |
| **Outpatient and day** | | | |
| Audiology outpatient | £117.39 | Per attendance | NHS reference costs 2019/2020 updated to 2021 prices. |
| Cardiology outpatient | £146.33 | Per attendance | NHS reference costs 2019/2020 updated to 2021 prices. |
| Clinical haematology outpatient | £170.66 | Per attendance | NHS reference costs 2019/2020 updated to 2021 prices. |
| Clinical immunology outpatient | £306.74 | Per attendance | NHS reference costs 2019/2020 updated to 2021 prices. |
| Colonoscopy day case | £684.21 | Per day case | NHS reference costs 2019/2020 updated to 2021 prices (weighted average of all descriptions citing ‘colonoscopy’). |
| CT scan | £97.93 | Per attendance | NHS reference costs 2019/2020 updated to 2021 prices (weighted average of all descriptions citing 'computerised tomography'). |
| Day case average | £824.52 | Per day case | NHS reference costs 2019/2020 updated to 2021 prices (weighted average of all descriptions). |
| Dermatology outpatient | £124.74 | Per attendance | NHS reference costs 2019/2020 updated to 2021 prices. |
| Dietician outpatient | £94.57 | Per attendance | NHS reference costs 2019/2020 updated to 2021 prices. |
| ECG | £162.12 | Per attendance | NHS reference costs 2019/2020 updated to 2021 prices. |
| Sleep clinic | £188.64 | Per day case | NHS reference costs 2019/2020 updated to 2021 prices. |
| Endocrinology outpatient | £166.40 | Per attendance | NHS reference costs 2019/2020 updated to 2021 prices. |
| ENT outpatient | £115.43 | Per attendance | NHS reference costs 2019/2020 updated to 2021 prices. |
| Gastroenterology day case | £594.63 | Per day case | NHS reference costs 2019/2020 updated to 2021 prices (weighted average of all descriptions citing 'gastrointestinal', excluding those aged ≤18 years). |
| Gastroenterology outpatient | £149.81 | Per attendance | NHS reference costs 2019/2020 updated to 2021 prices. |
| General surgery outpatient | £144.04 | Per attendance | NHS reference costs 2019/2020 updated to 2021 prices. |
| Gynaecology day case | £575.57 | Per day case | NHS reference costs 2019/2020 updated to 2021 prices (weighted average of all descriptions citing 'gynaecological'). |
| Gynaecology outpatient | £154.54 | Per attendance | NHS reference costs 2019/2020 updated to 2021 prices. |
| MRI | £178.72 | Per attendance | NHS reference costs 2019/2020 updated to 2021 prices. |
| Mastectomy day case | £3,718.24 | Per day case | NHS reference costs 2019/2020 updated to 2021 prices (weighted average of all 'Bilateral Major Breast Procedures with Lymph Node Clearance' or 'Unilateral Major Breast Procedures with Lymph Node Clearance' descriptions). |
| Medical oncology outpatient | £198.79 | Per attendance | NHS reference costs 2019/2020 updated to 2021 prices. |
| Neurology outpatient | £198.15 | Per attendance | NHS reference costs 2019/2020 updated to 2021 prices. |
| Ophthalmology outpatient | £111.03 | Per attendance | NHS reference costs 2019/2020 updated to 2021 prices. |
| Orthopaedics outpatient | £125.66 | Per attendance | NHS reference costs 2019/2020 updated to 2021 prices. |
| Other diagnostic imaging | £66.61 | Per unit | NHS reference costs 2019/2020 updated to 2021 prices. |
| Outpatient average | £139.76 | Per attendance | NHS reference costs 2019/2020 updated to 2021 prices. |
| Pain management outpatient | £187.23 | Per attendance | NHS reference costs 2019/2020 updated to 2021 prices. |
| Phlebotomy | £3.78 | Per unit | NHS reference costs 2019/2020 updated to 2021 prices. |
| Physiotherapy outpatient | £64.38 | Per attendance | NHS reference costs 2019/2020 updated to 2021 prices. |
| Plastic surgery outpatient | £120.95 | Per attendance | NHS reference costs 2019/2020 updated to 2021 prices. |
| PFT | £150.78 | Per attendance | NHS reference costs 2019/2020 updated to 2021 prices. |
| Respiratory medicine outpatient | £161.07 | Per attendance | NHS reference costs 2019/2020 updated to 2021 prices. |
| Rheumatology outpatient | £150.86 | Per attendance | NHS reference costs 2019/2020 updated to 2021 prices. |
| Ultrasound | £48.79 | Per unit | NHS reference costs 2019/2020 updated to 2021 prices. |
| Urology outpatient | £113.84 | Per unit | NHS reference costs 2019/2020 updated to 2021 prices. |
| X-ray | £29.50 | Per unit | NHS reference costs 2019/2020 updated to 2021 prices (diagnostic imaging - direct access - plain film). |
| **A&E** | | | |
| Ambulance admitted | £621.87 | Per attendance | NHS reference costs 2019/2020 updated to 2021 prices (Ambulance (see hear treat and convey) & weighted average of admitted AE, excluding dental and dead-on arrival). |
| No ambulance admitted | £320.78 | Per attendance | NHS reference costs 2019/2020 updated to 2021 prices (weighted average of admitted AE, excluding dental and dead-on arrival). |
| Ambulance not admitted | £461.15 | Per attendance | NHS reference costs 2019/2020 updated to 2021 prices (Ambulance (see hear treat and convey) & weighted average of not admitted AE, excluding dental and dead-on arrival). |
| No ambulance not admitted | £160.06 | Per attendance | NHS reference costs 2019/2020 updated to 2021 prices (weighted average of not admitted AE, excluding dental and dead-on arrival). |
| **Primary, community and social care** | | | |
| Cardiac rehabilitation outpatient | £109.47 | Per attendance | NHS reference costs 2019/2020 updated to 2021 prices. |
| Diabetic nurse | £81.19 | Per attendance | NHS reference costs 2019/2020 updated to 2021 prices (Specialist Nursing, Diabetic Nursing/Liaison, Adult, Face to face). |
| Dietician outpatient attendance | £94.57 | Per attendance | NHS reference costs 2019/2020 updated to 2021 prices. |
| General dental service attendance | £113.65 | Per attendance | NHS reference costs 2019/2020 updated to 2021 prices. |
| GP consultation | £33.00 | Per consultation | PSSRU Unit Costs of Health and Social Care 2021. Community based nurses, doctors and dentists. |
| GP telephone consultation | £15.52 | Per consultation | PSSRU Unit Costs of Health and Social Care 2020 (10.5 Telephone triage – GP-led and nurse-led). |
| Gynaecology outpatient | £154.62 | Per attendance | NHS reference costs 2019/2020 updated to 2021 prices. |
| Home care | £24 | Per hour | PSSRU Unit Costs of Health and Social Care 2021. Community based social care professionals. |
| Hospice support | £116.57 | Per unit | NHS reference costs 2019/2020 updated to 2021 prices (Specialist Nursing, Palliative/Respite Care, Adult, Face to face). |
| Long COVID clinic | £158.63 | Per attendance | NHS reference costs 2019/2020 updated to 2021 prices. |
| Nurse consultation | £10.85 | Per consultation | PSSRU Unit Costs of Health and Social Care 2021. Community based nurses (nurse GP practice). |
| Nurse telephone consultation | £7.80 | Per consultation | PSSRU Unit Costs of Health and Social Care 2020 (10.5 Telephone triage – GP-led and nurse-led). |
| Occupational therapy outpatient | £74.79 | Per attendance | NHS reference costs 2019/2020 updated to 2021 prices. |
| Optometry outpatient | £93.52 | Per attendance | NHS reference costs 2019/2020 updated to 2021 prices. |
| Phlebotomy | £3.78 | Per unit | NHS reference costs 2019/2020 updated to 2021 prices. |
| Physiotherapy outpatient | £64.38 | Per attendance | NHS reference costs 2019/2020 updated to 2021 prices. |
| Cervical screening | £34.84 | Per unit | NHS reference costs 2019/2020 updated to 2021 prices (Special Screening, Examinations or Other Genetic Disorders). |
| Social worker | £46.00 | Per hour | PSSRU Unit Costs of Health and Social Care 2021.Community based social care professionals (social worker; adult services). |
| Speech and language therapy outpatient | £108.54 | Per attendance | NHS reference costs 2019/2020 updated to 2021 prices. |
| X-ray | £29.50 | Per unit | NHS reference costs 2019/2020 updated to 2021 prices (diagnostic imaging - imaging: direct Access - plain film). |
| **Notes:** Where multiple relevant costs or bands are available, weighted averages have been used. Unless otherwise stated mental health support contacts were assumed to be 1 hour in duration. In the mental health section, if data were missing or insufficient, the cost was set to missing due to the wide variation in mental health support services accessed. Due to the limited use and uncertainty in the costings of private services, these have been costed using the same unit costs as NHS and social care. Where stated costs have been updated using the NHS cost inflation index (NHSCII). ^a^The form was not designed to collect medication data, however some participants did report mental health medication costs most typically referring to sertraline, therefore to simply cost this a 6-month supply of sertraline using the most common daily dose was applied. | | | |

Table 3. N (%) for data pertaining to Hub contact and mental health support access following the completion of Hub screening.

|  | | **Site A**  **(n = 77)** | **Site B**  **(n = 29)** | **Site C**  **(n = 46)** | **Site D**  **(n = 147)** | **Total**  **(N = 299)** |
| --- | --- | --- | --- | --- | --- | --- |
| **Further contact with Hub staff** | | | | | | |
| Contact of any kind | Yes | 62 (80.5) | 21 (72.4) | 37 (80.4) | 99 (67.4) | 219 (73.2) |
|  | No | 14 (18.2) | 7 (24.1) | 6 (13.0) | 42 (28.6) | 69 (23.1) |
|  | Don’t Know | 1 (1.3) | 1 (3.5) | 1 (2.2) | 6 (4.1) | 9 (3.0) |
|  | Missing | *0% missing* | *0% missing* | *4% missing* | *0% missing* | *1% missing* |
| Telephone | Yes | 62 (80.5) | 21 (72.4) | 32 (69.6) | 91 (61.9) | 206 (68.9) |
|  | No | 0 | 0 | 4 (8.7) | 8 (5.4) | 12 (4.0) |
|  | Don’t Know | 0 | 0 | 1 (2.2) | 0 | 1 (0.3) |
|  | Missing | *19%missing* | *28% missing* | *20% missing* | *33% missing* | *27% missing* |
| Email | Yes | 33 (42.9) | 13 (44.8) | 30 (65.2) | 56 (38.1) | 132 (44.2) |
|  | No | 25 (32.5) | 6 (20.7) | 6 (13.0) | 39 (26.5) | 76 (25.4) |
|  | Don’t Know | 3 (3.9) | 2 (6.9) | 0 | 4 (2.7) | 9 (3.0) |
|  | Missing | *21% missing* | *28% missing* | *22% missing* | *33% missing* | *27% missing* |
| **Access to mental health support** | | | | | | |
| via any route | Yes | 63 (81.8) | 20 (69.0) | 23 (50.0) | 65 (44.2) | 171 (57.2) |
|  | No | 13 (16.9) | 8 (27.6) | 20 (43.5) | 72 (49.0) | 113 (37.8) |
|  | *Missing* | *1% missing* | *3% missing* | *6% missing* | *7% missing* | *5% missing* |
| Provided by the Hub | Yes | 51 (81.0) | 13 (65.0) | 15 (65.2) | 16 (24.6) | 95 (55.6) |
|  | No | 11 (17.5) | 7 (35.0) | 6 (26.1) | 45 (69.2) | 69 (40.4) |
|  | Missing | 2% missing | 0% missing | 9% missing | 6% missing | 4% missing |
| Provided by employer | Yes | 5 (7.9) | 1 (5.0) | 2 (8.7) | 13 (20.0) | 21 (12.3) |
|  | No | 13 (20.6) | 8 (40.0) | 10 (43.5) | 37 (56.9) | 68 (39.8) |
|  | *Missing* | *71% missing* | *55% missing* | *48% missing* | *23% missing* | *48% missing* |
| Non-hub support that was accessed by direct support of the hub | Yes | 5 (7.9) | 4 (20.0) | 4 (17.4) | 19 (29.2) | 32 (18.7) |
|  | No | 51 (81.0) | 13 (65.0) | 15 (65.2) | 16 (24.6) | 95 (55.6) |
|  | *Missing* | *11% missing* | *15% missing* | *17% missing* | *46% missing* | *26% missing* |
| **Waiting list for mental health support** | | | | | | |
| via any route | Yes | 12 (15.6) | 1 (3.5) | 9 (19.6) | 12 (8.2) | 34 (11.4) |
|  | No | 62 (80.5) | 25 (86.2) | 32 (69.6) | 121 (82.3) | 240 (80.3) |
|  | *Missing* | *4% missing* | *10% missing* | *11% missing* | *9% missing* | *8% missing* |
| Provided by the Hub | Yes | 2 (16.7) | 0 | 6 (66.7) | 0 | 8 (23.5) |
|  | No | 9 (75.0) | 1 (100) | 2 (22.2) | 12 (100) | 24 (70.6) |
|  | *Missing* | *8% missing* | *0% missing* | *11% missing* | *0% missing* | *6% missing* |
| Provided by employer | Yes | 0 | 0 | 0 | 2 (16.7) | 2 (5.9) |
|  | No | 9 (75.0) | 1 (100) | 2 (22.2) | 8 (66.7) | 20 (58.8) |
|  | *Missing* | *25% missing* | *0% missing* | *78% missing* | *17% missing* | *35% missing* |
| Non-hub support that was accessed by direct support of the hub | Yes | 3 (25.0) | 1 (100) | 0 | 6 (50.0) | 10 (29.4) |
|  | No | 2 (16.7) | 0 | 6 (66.7) | 0 | 8 (23.5) |
|  | *Missing* | *58% missing* | *0% missing* | *33% missing* | *50% missing* | *47% missing* |
